# Delayed hypersensitivity reaction to orodispersible budesonide in a case with eosinophilic esophagitis

**DOI:** 10.1186/s12876-020-01554-y

**Published:** 2020-12-11

**Authors:** Caroline Michèle Andrist, Lukas Jörg, Thomas Greuter, Anna Gschwend, Alex Straumann, Arthur Helbling

**Affiliations:** 1 Division of Allergology, Department of Rheumatology, Immunology, and Allergology, Inselspital, Bern University Hospital, 3010 Bern, Switzerland; 2 Department of Gastroenterology and Hepatology, University Hospital Zurich, Zurich, Switzerland; 3 Swiss EoE Clinics and Research Network, Department of Gastroenterology and Hepatology, University Hospital Zurich, Zurich, Switzerland

**Keywords:** Orodispersible budesonide, Delayed hypersensitivity reaction, Eosinophilic esophagitis, Jorveza, Exanthema

## Abstract

**Background:**

Eosinophilic esophagitis (EoE) is a chronic inflammatory disease that has been known since the early 1990s. Swallowed topical corticosteroids (STC) belong to the therapeutic cornerstones. We describe a delayed hypersensitivity reaction to Jorveza®, a newly developed orodispersible budesonide tablet licensed for the treatment of eosinophilic esophagitis.

**Case presentation:**

A 32-year-old Caucasian woman with EoE was newly treated with Jorveza®. Hours after the first intake, she felt a “strange pruritus” in the throat. This sensation worsened with each subsequent intake. On day 4 she developed oral mucosal symptoms (paresthesia of the tongue, sore and an itchy throat). Intraoral, throat and facial swellings, but no systemic reaction were observed. Patch testing using two commercial test series as well as the orodispersible budesonide tablet revealed a strong sensitization, proving a T cell mediated allergy to budesonide.

**Conclusions:**

Orodispersible budesonide is increasingly prescribed for the treatment of eosinophilic esophagitis. The development of oropharyngeal symptoms after initiating should alert the treating physician to the possibility of a hypersensitivity reaction.

## Background

Eosinophilic esophagitis (EoE) is an esophageal-restricted, chronic inflammatory disease often associated with atopy. The prevalence of EoE is dramatically increasing and has currently reached values about 43 individuals per 100,000 inhabitants [1]. EoE occurs in people of all ages [2], but is more prevalent among men and more common in Caucasians than in Africans [3]. Current therapeutic opinions are built on the *three Ds*: *D*rugs (swallowed topical corticosteroids [STC], proton-pump inhibitors [PPIs] and biologics), *D*ietary restrictions to avoid alimentary antigens and, if the disease has led to the formation of strictures in advanced stages, endoscopic *D*ilation [4]. The effectiveness of STCs as induction treatment for acute EoE is well documented by a multitude of randomized controlled trials [5, 6]. The effect can be explained by a reduction of inflammatory cells, which leads to less fibrosis and also reverses IL13-induced pathways [7].

Among STCs, fluticasone and budesonide are the most commonly used in the management of EoE [7]. Both are topically effective but have to be swallowed for the treatment. In January 2018, the orodispersible budesonide tablet Jorveza® (budesonide, Dr. Falk Pharma GmbH, Freiburg, Germany) was approved by European Medicines Agency for the treatment of EoE, however, to date there is still no FDA approval. This orodispersible budesonide tablet has a special galenic formulation designed to act locally at the site of inflammation in the esophagus [8]. Over a period of several minutes, the melting tablet dissolves in the mouth and the effervescent corpuscles contained in it stimulate the flow of saliva, which, with its adhesive properties, is an ideal medium for the active ingredient, as it transports the dissolved budesonide to the esophageal mucosa when swallowed, where it can exert its effects locally [8].

## Case presentation

A 32-year-old Caucasian woman with atopy and relevant seasonal allergic rhinitis in the springtime was diagnosed EoE in 09/2018. Symptoms were somewhat atypical with postprandial coughing, especially after consumption of pasta and bread, regurgitation and intermittent dyspnea. After diagnosing EoE, treatment with fluticasone powder was initiated and continued for 16 months. Induction therapy was started at 2x1000μg/day for 2 weeks, then reduced to 2x500μg/day and 3x250μg/day, respectively. This therapy with fluticasone was in off-label prescription. When the orodispersible budesonide tablet became available in Switzerland, a change in therapy was planned. The switch to orodispersible budesonide 2x1mg/day is called day 1. Approximately 8 hours after the intake of the first effervescent tablet, a “strange pruritus” was felt in the throat. This sensation worsened with each subsequent intake. Three days later (day 4) approximately 1 h after the intake of the evening dose symptoms progressed and the patient complained of dysphagia and dysarthria. On day 5, she consulted an otolaryngologist office because of persisting tongue-numbness and a burning throat sensation. In the evening of day 5 tongue, lips, face and the oral mucosa became swollen. She was unable to lie down because of swallowing difficulties due to increased mucus production. She felt a burning pain behind the sternum and experienced a feeling of tightness. In the morning of day 6, she was referred to the emergency unit of the local hospital. The orodispersible budesonide was stopped. Clinical examination showed a generalized intraoral swelling and deep mucosal redness. In addition, small transparent vesicles grouped in the middle of the hard palate were noticed. A smear test of the vesicles on the palate was positive for *H. simplex* virus type 1. CRP was slightly elevated (10,9 mg/l), eosinophil count was normal (0.30G/l).

After treatment with clemastine (3 mg) and methylprednisolone 40 mg i.v. facial and mucosal swelling subsided within a few hours. A consecutive oral therapy with cetirizine 10 mg was prescribed. Swelling of the upper lips reoccurred the next day (day 7), but symptoms disappeared with oral prednisolone 50 mg/day over a period of 2 days. Dose was tapered to 20 mg and finally to 10 mg/day over a week. This dose was maintained for 2 weeks. Because allergy to budesonide was suspected, the patient was referred to an allergy workup.

**Fig. 1:**
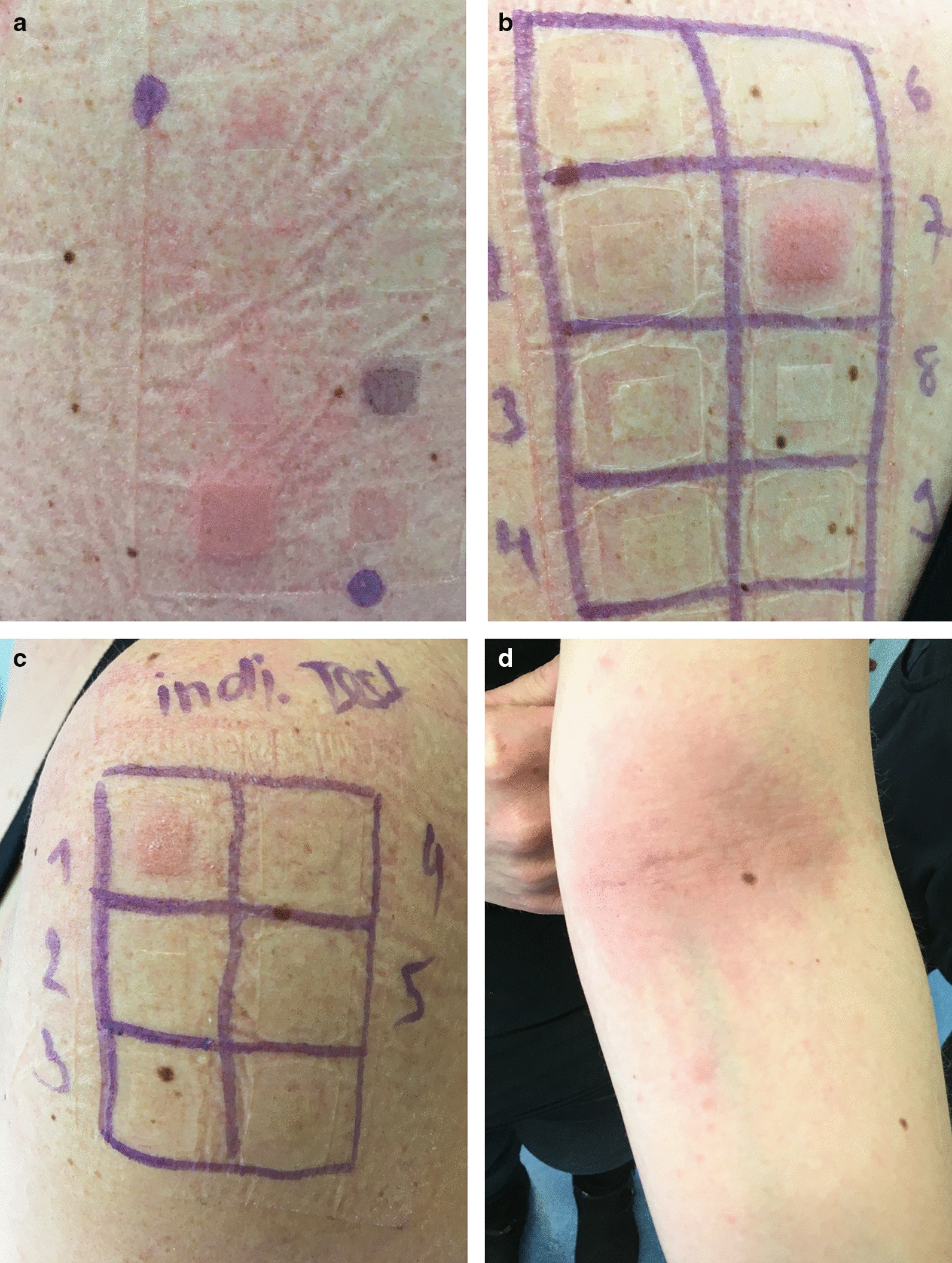
48 h after application of patch tests significantly positive reactions (++) were observed with erythema, papules (vesicules) to (**a**) budesonide on panel 3 of the T.R.U.E. TEST®, **b** budesonide on the test series allergEAZE®, **c** Jorveza®. One day after test application, mild exanthemas with involvement of both elbows, neck and décolleté area were observed. The left elbow is shown here (**d**)

The allergy workup was performed within 2 months after the reaction and included patch tests to Jorveza® and the corticosteroids tixocortol pivalate 1%, hydrocortisone 1%, prednisolone 1%, budesonide 0.1%, amicinonide 0.1%, triamcinolonacetonide 0.1%, dexamethasone 0.1%, hydrocortisone-17-butyrate 0.1%, betametasone-17-valerate 0.12% and clobetasol-17-propionate 0.25% and tixocortol-21-pivalate 3 μg/cm^2^, budesonide 1 μg/cm^2^, hydrocortizone-17-butyrate 20 μg/cm^2^ (allergEAZE® and T.R.U.E. TEST® SmartPractice, Hillerod, Denmark). Petrolatum was used as a negative control. Patch tests were removed 48 h after application and skin reactions were read after 48 and 72 h. Significantly positive reactions (++) with erythematous papules and isolated vesicules were observed to budesonide (tested twice) (Fig. 1(a), (b)) and the incriminated medication Jorveza® (Fig. 1(c)). Overnight after test application, a mild itchy flexural exanthema involving the insight of both elbows, neck, behind the ears and the décolleté area developed (Fig. 1(d)).

Since the patient subsequently developed coughing and dysphagia again as before EoE treatment, the therapy was switched back to the formerly well tolerated fluticasone on day 45 with 500 μg/day for 5 days following which the dose was raised to 1000 μg/day. No side effects have been observed since.

Retrospectively, the patient was already treated with inhaled budesonide (Symbicort®, AstraZeneca AB, Sweden) 17 years ago for 6 months, but did not develop any symptoms at that time.

## Discussion and conclusions

To the best of our knowledge, this is the first report of a delayed hypersensitivity reaction to Jorveza®. Jorveza® a budesonide formulation specifically designed for the esophageal administration is the first drug approved by the regulatory authorities for the medical treatment of EoE and is now increasingly used for this indication.

Allergies to budesonide are rare, but have been published in the literature [9]. Budesonide causes mostly delayed type hypersensitivity reactions as experienced by our patient. Maybe a reactivation of the herpes simplex virus has triggered the hypersensitivity reaction to budesonide by T cells. Nevertheless, patch tests revealed a clear-cut monosensitization to budesonide and the orodispersible budesonide tablet, respectively. How sensitization to budesonide occurred remains open. Based on the patient’s history, inhaled budesonide was prescribed years before.

Following the index-reaction, the patient did tolerate prednisolone, topical clobetasole and fluticasone (Gr B). Therefore, except budesonide, CS for a systemic or topical therapy are available as well as fluticasone for the EoE treatment.

In conclusion, even though STCs are the most effective drugs to treat EoE efficiently, allergic reactions to STCs may hamper an optimal therapeutic course. When developing oropharyngeal symptoms after the onset of Jorveza®, a hypersensitivity reaction must be considered.

## Data Availability

The data used on this case reoprt is available from the corresponding author on reasonable request.

## Abbreviations

EoE: Eosinophilic esophagitis
STC: Swallowed topical corticosteroids
CS: Corticosteroids
